# 5,6,7,8-Tetra­hydro­naphthalene-1-carboxylic acid

**DOI:** 10.1107/S1600536809049721

**Published:** 2009-11-28

**Authors:** Pei Zou, Min-Hao Xie, Hao Wu, Ya-Ling Liu, Yong-Jun He

**Affiliations:** a Jiangsu Institute of Nuclear Medicine, Wuxi 214063, People’s Republic of China

## Abstract

In the mol­ecule of the title compound, C_11_H_12_O_2_, the cyclo­hexane ring adopts a half-chair conformation. In the crystal structure, mol­ecules are linked into centrosymmetric dimers by pairs of O—H⋯O hydrogen bonds, and the dimers are linked together by π–π inter­actions [centroid–centroid distance = 3.8310 (13) Å] and C—H⋯O bonds.

## Related literature

The title compoundis an inter­mediate in the synthesis of Palonosetron, a 5-HT_3_ receptor antagonist, see: Kowalczyk & Dvorak (1996 ▶); Lancelot *et al.* (1985 ▶). For bond-length data, see: Allen *et al.* (1987 ▶). 
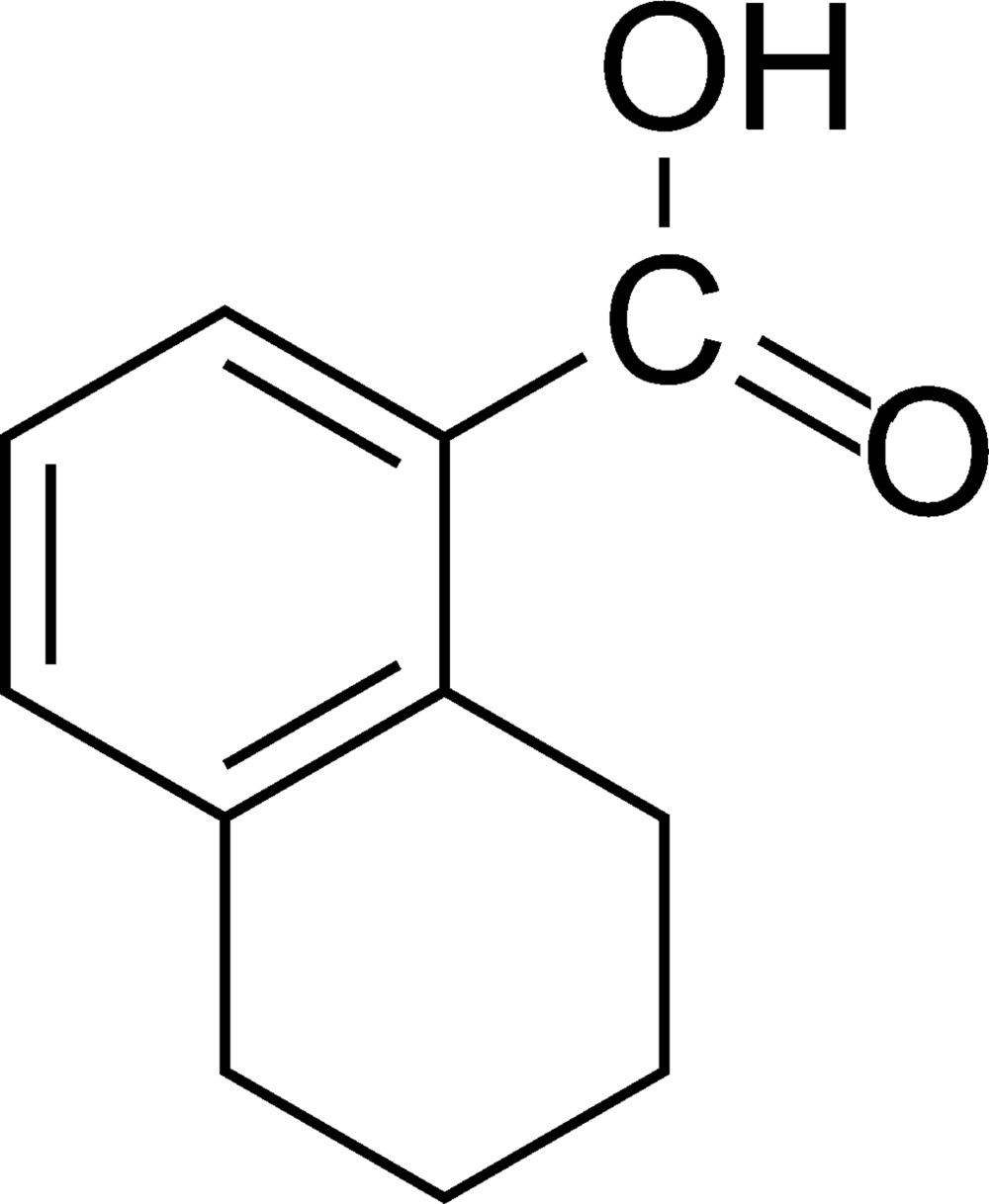


## Experimental

### 

#### Crystal data

C_11_H_12_O_2_
*M* *_r_* = 176.21
Triclinic, 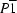

*a* = 7.477 (2) Å
*b* = 7.664 (2) Å
*c* = 8.546 (2) Å
α = 68.390 (10)°
β = 80.666 (12)°
γ = 75.977 (10)°
*V* = 440.3 (2) Å^3^
*Z* = 2
Mo *K*α radiation
μ = 0.09 mm^−1^
*T* = 93 K
0.27 × 0.23 × 0.12 mm

#### Data collection

Rigaku SPIDER diffractometer
Absorption correction: ψ scan (North *et al.*, 1968 ▶) *T*_min_ = 0.976, *T*_max_ = 0.989
4408 measured reflections
1994 independent reflections
1429 reflections with *I* > 2σ(*I*)
*R*_int_ = 0.026

#### Refinement

*R*[*F* ^2^ > 2σ(*F* ^2^)] = 0.039
*wR*(*F* ^2^) = 0.106
*S* = 0.97
1994 reflections
119 parameters
H-atom parameters constrained
Δρ_max_ = 0.38 e Å^−3^
Δρ_min_ = −0.17 e Å^−3^

### 

Data collection: *RAPID-AUTO* (Rigaku, 2004 ▶); cell refinement: *RAPID-AUTO*; data reduction: *RAPID-AUTO*; program(s) used to solve structure: *SHELXS97* (Sheldrick, 2008 ▶); program(s) used to refine structure: *SHELXL97* (Sheldrick, 2008 ▶); molecular graphics: *SHELXTL* (Sheldrick, 2008 ▶); software used to prepare material for publication: *SHELXTL*.

## Supplementary Material

Crystal structure: contains datablocks I, global. DOI: 10.1107/S1600536809049721/fk2006sup1.cif

Structure factors: contains datablocks I. DOI: 10.1107/S1600536809049721/fk2006Isup2.hkl

Additional supplementary materials:  crystallographic information; 3D view; checkCIF report

## Figures and Tables

**Table 1:** Hydrogen-bond geometry (Å, °)

*D*—H⋯*A*	*D*—H	H⋯*A*	*D*⋯*A*	*D*—H⋯*A*
O1—H10⋯O2^i^	0.84	1.80	2.6338 (15)	175
C8—H8⋯O2^ii^	0.95	2.58	3.509 (2)	165

Symmetry codes: (i) 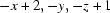; (ii) 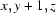.

**Fig. 1.**
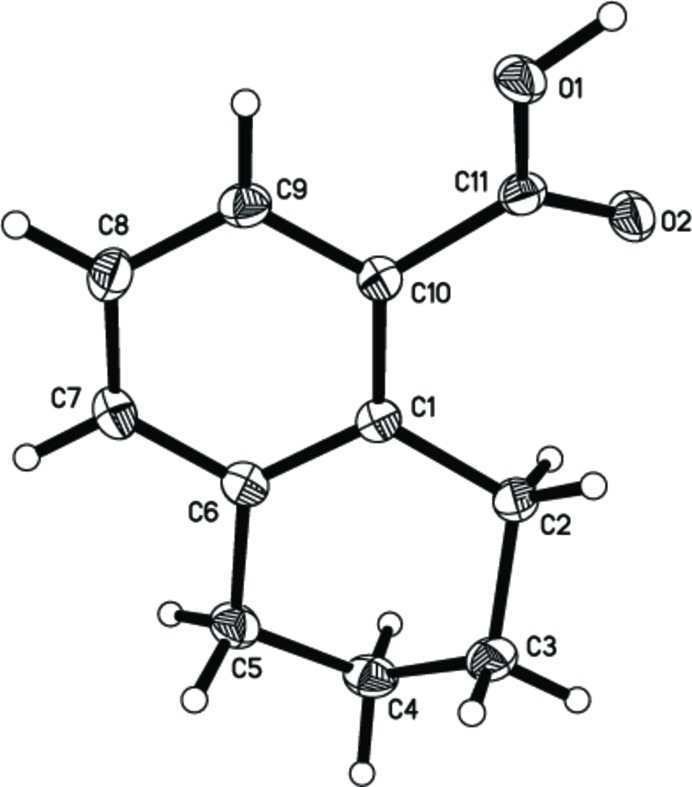
A view of the title compound with the atomic numbering scheme. Displacement ellipsoids were drawn at the 50% probability level.

**Fig. 2.**
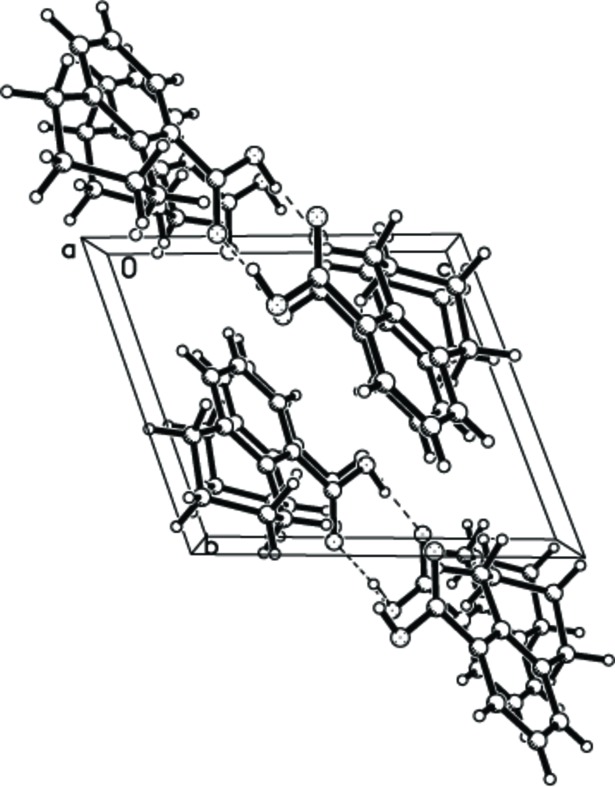
A packing diagram viewed along the a axis. Hydrogen bridging bonds are drawn as dashed lines.

## supplementary crystallographic information

### Comment

The title compound, (I), is useful as an intermediate in the synthesis of Palonosetron, a 5-HT_3_ receptor antagonists (Kowalczyk *et al.*, 1996; Lancelot *et al.*, 1985). We report here the crystal structure of (I), which is of interest to us in the field. The molecular structure is shown in Fig.1. The bond lengths and angles are within normal ranges (Allen *et al.*, 1987). The cyclohexane ring adopts a half chair conformation, with C3 lying out of the plane of the molecule by approximately 0.5 Å. In the crystal structure, intermolecular O—H···O hydrogen bonds (Tab. 1) link the molecules into centrosymmetric dimers(Fig. 2). Stacking of these dimers follows the π-π interactions, with the centroid-centroid distance of 3.8310 (13)Å [symmetry code(i): 1 - *x*, 1 - *y*, 1 - *z*].

### Experimental

A sample of commercial 5,6,7,8-Tetrahydronaphthalene-1-carboxylic acid (Aldrich) was crystalized by slow evaporation of a solution in methanol.

### Refinement

Positional parameters of all the H atoms bonded to C atoms were calculated geometrically and were allowed to ride on the C atoms to which they are bonded, with C—H=0.95 and 0.99 Å for aromatic and methylene and with *U*_iso_(H) = 1.2*U*_eq_(aromatic,methylene) parent atoms. H atom of the carboxyl group was derived from Fourier map, and constrained to ride on the parent atom with O—H=0.84 Å and *U*_iso_(H)=1.5*U*_eq_(O).

### Crystal data

**Table:**

C_11_H_12_O_2_	*Z* = 2
*M**_r_* = 176.21	*F*(000) = 188
Triclinic, *P*1	*D*_x_ = 1.329 Mg m^−^^3^
Hall symbol: -P 1	Mo *K*α radiation, λ = 0.71073 Å
*a* = 7.477 (2) Å	Cell parameters from 1274 reflections
*b* = 7.664 (2) Å	θ = 3.2–27.5°
*c* = 8.546 (2) Å	µ = 0.09 mm^−^^1^
α = 68.39 (1)°	*T* = 93 K
β = 80.666 (12)°	Block, colorless
γ = 75.977 (10)°	0.27 × 0.23 × 0.12 mm
*V* = 440.3 (2) Å^3^	

### Data collection

**Table:**

Rigaku SPIDER diffractometer	1994 independent reflections
Radiation source: fine-focus sealed tube	1429 reflections with *I* > 2σ(*I*)
graphite	*R*_int_ = 0.026
ω scans	θ_max_ = 27.5°, θ_min_ = 3.2°
Absorption correction: ψ scan (North *et al.*, 1968)	*h* = −9→9
*T*_min_ = 0.976, *T*_max_ = 0.989	*k* = −9→9
4408 measured reflections	*l* = −8→11

### Refinement

**Table:**

Refinement on *F*^2^	Primary atom site location: structure-invariant direct methods
Least-squares matrix: full	Secondary atom site location: difference Fourier map
*R*[*F*^2^ > 2σ(*F*^2^)] = 0.039	Hydrogen site location: geom and difmap
*wR*(*F*^2^) = 0.106	H-atom parameters constrained
*S* = 0.97	*w* = 1/[σ^2^(*F*_o_^2^) + (0.0575*P*)^2^] where *P* = (*F*_o_^2^ + 2*F*_c_^2^)/3
1994 reflections	(Δ/σ)_max_ < 0.001
119 parameters	Δρ_max_ = 0.38 e Å^−^^3^
0 restraints	Δρ_min_ = −0.17 e Å^−^^3^

### Special details

**Table:**

Geometry. All e.s.d.'s (except the e.s.d. in the dihedral angle between two l.s. planes) are estimated using the full covariance matrix. The cell e.s.d.'s are taken into account individually in the estimation of e.s.d.'s in distances, angles and torsion angles; correlations between e.s.d.'s in cell parameters are only used when they are defined by crystal symmetry. An approximate (isotropic) treatment of cell e.s.d.'s is used for estimating e.s.d.'s involving l.s. planes.
Refinement. Refinement of *F*^2^ against ALL reflections. The weighted *R*-factor *wR* and goodness of fit *S* are based on *F*^2^, conventional *R*-factors *R* are based on *F*, with *F* set to zero for negative *F*^2^. The threshold expression of *F*^2^ > σ(*F*^2^) is used only for calculating *R*-factors(gt) *etc*. and is not relevant to the choice of reflections for refinement. *R*-factors based on *F*^2^ are statistically about twice as large as those based on *F*, and *R*- factors based on ALL data will be even larger.

### Fractional atomic coordinates and isotropic or equivalent isotropic displacement parameters (Å^2^)

**Table:**

	*x*	*y*	*z*	*U*_iso_*/*U*_eq_	
O1	0.92438 (13)	0.25317 (11)	0.46074 (12)	0.0193 (3)	
H10	1.0051	0.1691	0.4343	0.029*	
O2	0.81137 (13)	−0.00378 (12)	0.63071 (12)	0.0213 (3)	
C1	0.49946 (18)	0.26317 (17)	0.73643 (16)	0.0151 (3)	
C2	0.43087 (18)	0.09079 (18)	0.73976 (17)	0.0181 (3)	
H2A	0.4896	−0.0245	0.8290	0.022*	
H2B	0.4691	0.0707	0.6302	0.022*	
C3	0.22129 (19)	0.11404 (19)	0.77249 (18)	0.0214 (3)	
H3A	0.1617	0.2140	0.6736	0.026*	
H3B	0.1849	−0.0078	0.7889	0.026*	
C4	0.15578 (19)	0.17011 (19)	0.92902 (17)	0.0220 (3)	
H4A	0.0212	0.1744	0.9550	0.026*	
H4B	0.2199	0.0736	1.0270	0.026*	
C5	0.19689 (19)	0.36544 (19)	0.89793 (18)	0.0214 (3)	
H5A	0.1020	0.4652	0.8285	0.026*	
H5B	0.1864	0.3847	1.0077	0.026*	
C6	0.38656 (18)	0.39253 (18)	0.81011 (16)	0.0173 (3)	
C7	0.4472 (2)	0.55486 (18)	0.80077 (17)	0.0198 (3)	
H7	0.3701	0.6409	0.8518	0.024*	
C8	0.61567 (19)	0.59422 (18)	0.71966 (17)	0.0206 (3)	
H8	0.6535	0.7062	0.7141	0.025*	
C9	0.72881 (19)	0.46801 (18)	0.64642 (17)	0.0182 (3)	
H9	0.8449	0.4937	0.5899	0.022*	
C10	0.67299 (18)	0.30349 (17)	0.65533 (16)	0.0153 (3)	
C11	0.80592 (18)	0.16973 (18)	0.58206 (16)	0.0163 (3)	

### Atomic displacement parameters (Å^2^)

**Table:**

	*U*^11^	*U*^22^	*U*^33^	*U*^12^	*U*^13^	*U*^23^
O1	0.0163 (5)	0.0170 (5)	0.0231 (5)	−0.0046 (4)	0.0068 (4)	−0.0076 (4)
O2	0.0205 (5)	0.0159 (5)	0.0274 (6)	−0.0060 (4)	0.0067 (4)	−0.0092 (4)
C1	0.0155 (7)	0.0148 (6)	0.0146 (6)	−0.0036 (5)	−0.0024 (5)	−0.0038 (5)
C2	0.0161 (7)	0.0184 (7)	0.0203 (7)	−0.0059 (5)	0.0016 (6)	−0.0072 (6)
C3	0.0171 (7)	0.0214 (7)	0.0264 (8)	−0.0080 (5)	0.0002 (6)	−0.0071 (6)
C4	0.0155 (7)	0.0235 (7)	0.0236 (8)	−0.0062 (6)	0.0029 (6)	−0.0047 (6)
C5	0.0167 (7)	0.0240 (7)	0.0229 (7)	−0.0035 (5)	0.0040 (6)	−0.0099 (6)
C6	0.0162 (7)	0.0182 (7)	0.0159 (7)	−0.0025 (5)	−0.0002 (5)	−0.0050 (5)
C7	0.0208 (7)	0.0171 (7)	0.0207 (7)	−0.0008 (5)	0.0002 (6)	−0.0085 (6)
C8	0.0238 (8)	0.0159 (7)	0.0244 (8)	−0.0060 (5)	−0.0015 (6)	−0.0083 (6)
C9	0.0151 (7)	0.0190 (7)	0.0191 (7)	−0.0060 (5)	0.0007 (6)	−0.0044 (5)
C10	0.0158 (7)	0.0147 (6)	0.0147 (6)	−0.0026 (5)	−0.0008 (5)	−0.0047 (5)
C11	0.0140 (7)	0.0200 (7)	0.0165 (7)	−0.0060 (5)	−0.0003 (5)	−0.0067 (5)

### Geometric parameters (Å, °)

**Table:**

O1—C11	1.3254 (14)	C4—H4A	0.9900
O1—H10	0.8400	C4—H4B	0.9900
O2—C11	1.2307 (15)	C5—C6	1.5166 (18)
C1—C6	1.4049 (17)	C5—H5A	0.9900
C1—C10	1.4157 (18)	C5—H5B	0.9900
C1—C2	1.5182 (17)	C6—C7	1.3956 (18)
C2—C3	1.5247 (19)	C7—C8	1.3804 (19)
C2—H2A	0.9900	C7—H7	0.9500
C2—H2B	0.9900	C8—C9	1.3859 (18)
C3—C4	1.5242 (19)	C8—H8	0.9500
C3—H3A	0.9900	C9—C10	1.3941 (18)
C3—H3B	0.9900	C9—H9	0.9500
C4—C5	1.5196 (18)	C10—C11	1.4860 (18)
			
C11—O1—H10	109.5	C6—C5—H5A	108.7
C6—C1—C10	117.91 (12)	C4—C5—H5A	108.7
C6—C1—C2	119.92 (12)	C6—C5—H5B	108.7
C10—C1—C2	122.13 (11)	C4—C5—H5B	108.7
C1—C2—C3	112.64 (10)	H5A—C5—H5B	107.6
C1—C2—H2A	109.1	C7—C6—C1	119.70 (12)
C3—C2—H2A	109.1	C7—C6—C5	117.82 (11)
C1—C2—H2B	109.1	C1—C6—C5	122.47 (12)
C3—C2—H2B	109.1	C8—C7—C6	122.00 (12)
H2A—C2—H2B	107.8	C8—C7—H7	119.0
C4—C3—C2	110.24 (12)	C6—C7—H7	119.0
C4—C3—H3A	109.6	C7—C8—C9	119.04 (12)
C2—C3—H3A	109.6	C7—C8—H8	120.5
C4—C3—H3B	109.6	C9—C8—H8	120.5
C2—C3—H3B	109.6	C8—C9—C10	120.29 (12)
H3A—C3—H3B	108.1	C8—C9—H9	119.9
C5—C4—C3	109.55 (11)	C10—C9—H9	119.9
C5—C4—H4A	109.8	C9—C10—C1	121.05 (11)
C3—C4—H4A	109.8	C9—C10—C11	117.19 (12)
C5—C4—H4B	109.8	C1—C10—C11	121.71 (11)
C3—C4—H4B	109.8	O2—C11—O1	122.06 (11)
H4A—C4—H4B	108.2	O2—C11—C10	124.11 (11)
C6—C5—C4	114.16 (11)	O1—C11—C10	113.80 (11)
			
C6—C1—C2—C3	19.10 (17)	C6—C7—C8—C9	−0.5 (2)
C10—C1—C2—C3	−158.53 (13)	C7—C8—C9—C10	−0.2 (2)
C1—C2—C3—C4	−51.66 (15)	C8—C9—C10—C1	1.0 (2)
C2—C3—C4—C5	64.12 (14)	C8—C9—C10—C11	−176.76 (12)
C3—C4—C5—C6	−43.06 (16)	C6—C1—C10—C9	−1.0 (2)
C10—C1—C6—C7	0.30 (19)	C2—C1—C10—C9	176.64 (12)
C2—C1—C6—C7	−177.43 (12)	C6—C1—C10—C11	176.63 (12)
C10—C1—C6—C5	179.06 (12)	C2—C1—C10—C11	−5.7 (2)
C2—C1—C6—C5	1.3 (2)	C9—C10—C11—O2	151.98 (13)
C4—C5—C6—C7	−170.00 (12)	C1—C10—C11—O2	−25.8 (2)
C4—C5—C6—C1	11.22 (19)	C9—C10—C11—O1	−26.09 (18)
C1—C6—C7—C8	0.5 (2)	C1—C10—C11—O1	156.15 (12)
C5—C6—C7—C8	−178.34 (13)		

### Hydrogen-bond geometry (Å, °)

**Table:**

*D*—H···*A*	*D*—H	H···*A*	*D*···*A*	*D*—H···*A*
O1—H10···O2^i^	0.84	1.80	2.6338 (15)	175
C2—H2B···O2	0.99	2.48	2.8506 (19)	101
C8—H8···O2^ii^	0.95	2.58	3.509 (2)	165

Symmetry codes: (i) −*x*+2, −*y*, −*z*+1; (ii) *x*, *y*+1, *z*.

## References

[bb1] Allen, F. H., Kennard, O., Watson, D. G., Brammer, L., Orpen, A. G. & Taylor, R. (1987). *J. Chem. Soc. Perkin Trans. 2*, pp. S1–19.

[bb2] Kowalczyk, B. A. & Dvorak, C. A. (1996). *Synthesis*, **7**, 816–818.

[bb3] Lancelot, J. C., Rault, S., Laduree, D. & Robba, M. (1985). *Chem. Pharm. Bull.* **37**, 2798–2802.

[bb4] North, A. C. T., Phillips, D. C. & Mathews, F. S. (1968). *Acta Cryst.* A**24**, 351–359.

[bb5] Rigaku (2004). *RAPID-AUTO*. Rigaku Corporation, Tokyo, Japan.

[bb6] Sheldrick, G. M. (2008). *Acta Cryst.* A**64**, 112–122. 10.1107/S0108767307043930 18156677

